# Evaluation of Multiple-Choice Tests in Head and Neck Ultrasound Created by Physicians and Large Language Models

**DOI:** 10.3390/diagnostics15151848

**Published:** 2025-07-22

**Authors:** Jacob P. S. Nielsen, August Krogh Mikkelsen, Julian Kuenzel, Merry E. Sebelik, Gitta Madani, Tsung-Lin Yang, Tobias Todsen

**Affiliations:** 1 Department of Otorhinolaryngology, Head and Neck Surgery and Audiology, Copenhagen University Hospital (Rigshospitalet), 2100 Copenhagen, Denmark; jnie0904@regionh.dk (J.P.S.N.);; 2 Department of Clinical Medicine, University of Copenhagen, 2100 Copenhagen, Denmark; 3 Department of Otorhinolaryngology, Head and Neck Surgery, University Hospital Regensburg, 93053 Regensburg, Germany; 4 Department of Otolaryngology-Head and Neck Surgery, Emory University School of Medicine, Atlanta, GA 30332, USA; 5 The Winship Cancer Institute, Emory University, Atlanta, GA 30322, USA; 6 Imperial College Healthcare NHS Trust, London W6 8RF, UK; 7 Department of Otolaryngology, National Taiwan University Hospital, Taipei 100225, Taiwan; 8 Graduate Institute of Clinical Medicine, National Taiwan University College of Medicine, Taipei 100233, Taiwan; 9 CAMES—Copenhagen Academy for Medical Education and Simulation, Capital Region of Denmark, 2100 Copenhagen, Denmark

**Keywords:** ultrasound, multiple-choice quiz, LLM, head and neck, AI, learning

## Abstract

**Background/Objectives**: Otolaryngologists are increasingly using head and neck ultrasound (HNUS). Determining whether a practitioner of HNUS has achieved adequate theoretical knowledge remains a challenge. This study assesses the performance of two large language models (LLMs) in generating multiple-choice questions (MCQs) for head and neck ultrasound, compared with MCQs generated by physicians. **Methods**: Physicians and LLMs (ChatGPT, GPT4o, and Google Gemini, Gemini Advanced) created a total of 90 MCQs that covered the topics of lymph nodes, thyroid, and salivary glands. Experts in HNUS additionally evaluated all physician-drafted MCQs using a Delphi-like process. The MCQs were assessed by an international panel of experts in HNUS, who were blinded to the source of the questions. Using a Likert scale, the evaluation was based on an overall assessment including six assessment criteria: clarity, relevance, suitability, quality of distractors, adequate rationale of the answer, and an assessment of the level of difficulty. **Results**: Four experts in the clinical field of HNUS assessed the 90 MCQs. No significant differences were observed between the two LLMs. Physician-drafted questions (*n* = 30) had significant differences with Google Gemini in terms of relevance, suitability, and adequate rationale of the answer, but only significant differences in terms of suitability compared with ChatGPT. Compared to MCQ items (*n* = 16) validated by medical experts, LLM-constructed MCQ items scored significantly lower across all criteria. The difficulty level of the MCQs was the same. **Conclusions**: Our study demonstrates that both LLMs could be used to generate MCQ items with a quality comparable to drafts from physicians. However, the quality of LLM-generated MCQ items was still significantly lower than MCQs validated by ultrasound experts. LLMs are therefore cost-effective to generate a quick draft for MCQ items that afterward should be validated by experts before being used for assessment purposes. In this way, the value of LLM is not the elimination of humans, but rather vastly superior time management.

## 1. Introduction

There has been a notable increase in the use of surgeon-performed head and neck ultrasound by otolaryngologists—head and neck surgeons—in recent years [1,2,3]. The benefits include faster diagnostic workups, as patients do not need to wait for a referral for a neck ultrasound to a radiology department. However, as ultrasound is an operator-dependent imaging modality, it is essential to ensure competency among clinicians performing the ultrasound examinations [4]. To ensure a high standard of care, the European Federation of Ultrasound Societies in Medicine and Biology (EFSUMB) recommends competency-based training in head and neck ultrasound instead of using a fixed number of ultrasound examinations required for certification [5]. To ensure high competency in head and neck ultrasound, both theoretical knowledge and technical skills are essential. Currently, assessment tools exist for direct assessment of head and neck performance [6,7], while assessment tools for theoretical knowledge still need to be developed. Multiple-choice questions (MCQs) are recommended for this theoretical assessment [5]. These are often used to assess the theoretical knowledge of healthcare professionals [8]. The main advantage of MCQs is the high reliability per testing hour, which is primarily due to the fast answering time and allows comprehensive domain coverage [9]. In this way, MCQs are often integrated into the pre-graduate medical curriculum, such as the United States Medical Licensing Examination (USMLE) [10,11]. However, constructing a sufficiently large item bank of MCQs often requires extensive time and faculty resources, which presents a challenge [12].

Publicly available large language models (LLMs) such as Google Gemini and OpenAI’s ChatGPT [13,14] have been assessed in various medical fields, including the creation of MCQs [15,16,17,18,19,20]. LLMs theoretically hold the potential to create MCQs at a faster rate, but their quality should ideally be comparable to that produced by human medical experts. Previous studies on LLM MCQ creation have shown varying inaccuracies, ranging from 60 percent to one percent, depending on the model version. To date, most research has focused on earlier versions of the LLMs [20].

With recent advancements in LLM capabilities, we hypothesize that the latest models of Google Gemini (Gemini Advanced) and ChatGPT (GPT-4o) can be used to generate new MCQ items cost-effectively. To our knowledge, there has been no comparison among the models Gemini Advanced, GPT-4o, and human performance for MCQs in ultrasound competency assessment. This study aims to assess the performance of Google Gemini and ChatGPT in generating MCQs for head and neck ultrasound, comparing their output to items generated by physicians.

## 2. Materials and Methods

The study assesses a combination of 90 MCQs with answers created by either physicians or an LLM (ChatGPT or Google Gemini). The study covered three topics related to head and neck ultrasound: lymph nodes, thyroid, and salivary glands. For each topic, ten questions with answers were created for each of the three parts. To guarantee an equal comparison and recommendations, every question had a textual basis and three possible answers, where two were misleading and one was correct [21]. All questions were directly put into the evaluation module, randomized, and all tracks of the author to the questions were erased. No modification or interaction regarding clarity or phrasing was made.

### 2.1. MCQ Generation

#### 2.1.1. LLM-Generated MCQ

The version of ChatGPT used in this study was GPT-4o (version of May 2024), and the version of Google Gemini was Google Gemini Advanced (version of May 2024).

When utilizing the LLMs, the following prompt was used: “Please create a multiple-choice quiz with 10 questions aimed at evaluating a person’s expertise in performing ultrasound examinations [insert topic]. The questions should cover various levels of difficulty but must exclude topics related to ultrasound physics. For each question, please provide three answer options including one correct answer, and indicate the level of difficulty (Likert scale from 1–5)”.

After each prompt, a new section was made to reduce memory retention bias [22]. No revision or pre-screening of the LLM-generated MCQs was made before evaluation to assess their raw output and better understand the models’ unassisted capabilities.

#### 2.1.2. Physician-Generated MCQ

A medical student and an otolaryngologist-head and neck surgeon created MCQ test items (*n* = 30) based on theoretical insights from head and neck ultrasound textbooks and scientific literature [5,23,24,25,26,27]. All assessed test items focused on the topics of lymph nodes, thyroid, and salivary glands. Each question featured one single best answer accompanied by two distractors.

#### 2.1.3. Expert-Validated MCQ

An international panel of senior consultants in otolaryngology-head and neck surgery or diagnostic radiology, all of whom are experts in head and neck ultrasound, evaluated all physician-generated MCQ items in a Delphi-like process with the possibility to add extra items [28] (*n* = 16). Items that were considered essential by at least 80% of the experts were included in the final MCQ bank. If items received lower scores and the expert panel recommended improvements, the suggested changes would be implemented for reevaluation in the following Delphi round [29].

### 2.2. Question Assessment

**Figure 1:**
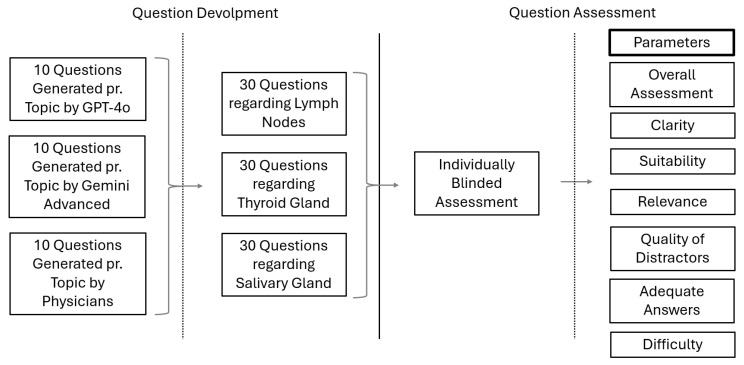
Figure showing the question development and assessment flow.

All the MCQ items were individually assessed by a group of experts blinded to whether human, ChatGPT, or Google Gemini generated the items. The experts consisted of an international group of senior consultants in diagnostic radiology or ENT with substantial experience in head and neck ultrasound. Each MCQ item was evaluated in terms of six assessment criteria: clarity, relevance, suitability, quality of distractors, adequate rationale of the answer, and an assessment of the level of difficulty. A Likert scale from one (worst) to ten (best) was used for each assessment (Supplementary File S1). Further, they also performed an overall assessment of the MCQ item using a Likert scale from one (worst) to five (best). All experts had the opportunity to supplement their answers with free-text comments. The assessment criteria were based on peer-reviewed literature to make it as unbiased as possible [30]. Study data were collected and managed using REDCap (Research Electronic Data Capture, version 15.0.21), a secure, web-based software platform hosted at Copenhagen University Hospital, Rigshospitalet [31]. The process is illustrated in Figure 1.

### 2.3. Statistical Analysis

Data analyses were performed using R (4.4.1 (14 June 2024) for macOS 12+; R Foundation for Statistical Computing, Vienna, Austria).

Mean scores and standard deviations (SD) were calculated for each assessment criterion. An analysis of the variance (ANOVA) was conducted to compare the internal variability between the two LLMs (Google Gemini and ChatGPT) and between each LLM and physician-generated MCQs. A *p*-value of 0.05 was considered significant.

## 3. Results

Four experts participated in assessing all 90 MCQs created by either physicians, ChatGPT, or Google Gemini. The experts consisted of one senior consultant in diagnostic radiology and three senior consultants in ENT, representing the USA, Europe, and Asia. All have more than 15 years’ experience in the clinical field of head and neck ultrasound.

Comparing the draft question MCQs from the physicians (*n* = 30) with the MCQs created by Google Gemini and ChatGPT, physician-drafted questions demonstrated higher mean scores across most of the assessment criteria compared with each of the two LLMs (Table 1).

**Figure 2:**
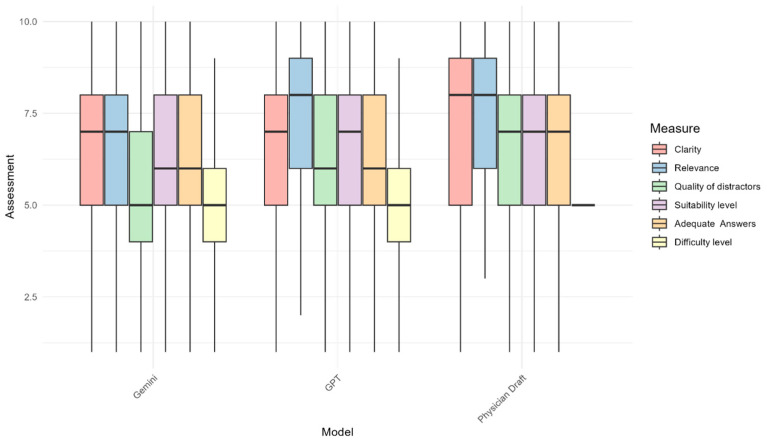
Boxplot illustrating the overall median, interquartile range, minimum, and maximum values for the assessment criteria across the evaluated entities: Google Gemini, ChatGPT, and Physicians Draft.

Physician-drafted questions had a significantly better mean score compared with Google Gemini in terms of relevance, suitability, and adequate rationale of the answer. However, the difference was only significantly better in terms of the suitability criterion when compared with ChatGPT. No significant difference was found between physicians and any of the LLMs in terms of the difficulty level of MCQs. Comparing Google Gemini and ChatGPT, no significant differences were observed between the two LLMs across any criteria. A comparison across all the assessment criteria and the evaluated entities—Google Gemini, ChatGPT, and physicians—is illustrated in Figure 2.

Assessing the MCQs that made it to the final item bank validated by medical experts (*n* = 16), they demonstrated statistically significantly higher mean scores compared with both Google Gemini and ChatGPT across all assessment criteria.

The usability of questions for multiple-choice quizzes was also assessed. The MCQs were categorized as (1) usable without modifications, (2) usable with modifications, and (3) unusable. Questions validated by medical experts had a usability rate of 64.1% (usable without modifications), whereas ChatGPT and Google Gemini showed lower usability rates, at 45.0% and 42.5%, respectively.

## 4. Discussion

We present the first comparison among the LLMs Google Gemini (Gemini Advanced), ChatGPT (GPT-4o), and physicians in the context of generating MCQs testing theoretical knowledge in ultrasound. Our results demonstrate that both LLM models can create MCQs with a quality score comparable to physician-generated items regarding all assessment criteria. However, their score was still significantly lower than the MCQ items validated by medical experts.

A strength of this study lies in the inclusion of two different LLMs, enabling an internal comparison of their performance. The use of draft questions generated by both physicians and expert-validated questions, which serve as a non-publicly available gold standard, further strengthens the study by providing a reliable benchmark for assessing question quality. By including questions across diverse topics in head and neck ultrasound, lymph nodes, thyroid, and salivary glands, the study demonstrates the broad applicability of its findings. Moreover, the evaluation by an international panel of experts, blinded to the question source, ensures unbiased assessments. However, the expert panel was also part of the committee that validated the physicians’ draft questions, introducing a potential bias. A limitation of our study is that the MCQs were purely text-based, and no ultrasound images or videos were used, since the LLMs cannot create or interpret a relevant clinical image without sufficient contextual information. Since ultrasound is a diagnostic discipline, it essentially involves the interpretation of images. In this way, images would be essential to incorporate into future studies. The models used in our study were public general LLMs, which is also a potential limitation, as head and neck ultrasound is a highly specialized area. The models would be likely to perform on a higher level if they were optimized for medical literature related to head and neck ultrasound. Another limitation of our study is that our research only demonstrates a momentary picture of the LLMs’ performance, which varies [32]. This is also a limitation in the usage of public general LLMs, which continue to evolve and, in turn, affect their output. Due to the diverse ways in which users prompt LLMs, the generated outputs often exhibit further variability [20]. An additional limitation is that we did not perform a statistical analysis of bias risk. As the experts were also involved in the Delphi-like process for MCQ generation, this may introduce a bias towards higher ratings of the physician-generated MCQ. However, as the experts were blinded to the source of the MCQ generation and had a period of more than one year between the generation of the MCQ and the Likert evaluations, we do not believe it would impact the results.

In contrast to our findings, a study by Mistry et al. using the same assessment parameters within radiology exam questions shows no difference in the scores between ChatGPT (version GPT-4) and MCQs created for the exam [30]. However, the exam MCQs used in their study were also publicly available online, meaning they could be part of the LLMs’ training material and, in this way, increase the performance. In comparison, the MCQs used in our study were not publicly available.

In another study, Cheung et al. provided reference material for the LLM and showcased almost identical mean scores across five different assessment criteria [19].

However, it should also be noted that this solution could have potential privacy issues, especially if the LLM is trained on proprietary or sensitive medical data. In addition, it is essential to consider that creating MCQs using LLMs may raise copyright issues, as the generated content might not meet the necessary criteria for human authorship [33].

Another important aspect in the creation of MCQs is the time spent creating the questions. This is often a time-consuming and thereby costly process [9]. In our study, both LLMs required less than 30 s to create 10 questions within the head and neck ultrasound topic. Cheung et al. also demonstrated that it took physicians significantly longer to create the MCQs compared with the LLM [19]. This time efficiency could have substantial resource allocation and cost benefits for training centers. This raises the question of whether the time and cost savings could justify some trade-off in quality. We would therefore suggest using LLMs to generate a first draft of MCQ items, of which our results show that almost half of them can be used directly in an MCQ. However, an expert validation is needed to ensure clinical accuracy and educational relevance. This “hybrid approach” could then impart some time savings when developing MCQs without compromising quality. These findings should be considered in the development of future medical curricula, as the time required to create MCQs can be significantly reduced with LLM, and MCQs can be integrated more effectively into teaching and knowledge assessment.

We also recognize the ethical implications of utilizing LLMs in medical education. These especially include potential biases [34]. We emphasize that LLMs’ use must be transparent and critically evaluated.

In conclusion, our study showed that both LLMs can create MCQs of the same quality as the draft questions created by physicians. However, the quality of LLM-generated MCQs was significantly lower than that of MCQs validated by ultrasound experts.

LLMs are therefore cost-effective for generating rapid initial draft MCQ items, which require further validation by experts before being used for assessment purposes.

For future perspectives, fine-tuned domain-specific LLMs, such as OpenEvidence or ChatRWD [35], could show potential to create MCQs within the field of medical education. This fine-tuning could be based on expert-generated questions to ensure quality and relevance. Further elaboration on the process, such as potential collaboration with institutions to develop secure, specialty-specific datasets, could create even greater potential. The implementation in a clinical workflow and financial aspects could be investigated in further studies.

## Figures and Tables

**Table 1:** Comparative scores across the two large language models and physicians’ draft questions.

	Gemini	GPT	Physician Draft	Gemini vs. GPT	Gemini vs. Physician Draft	GPT vs. Physician Draft
Measure	Mean (SD)	Mean (SD)	Mean (SD)	*p*-Value	*p*-Value	*p*-Value
Overall Assessment	2.7 (0.9)	2.8 (0.8)	2.9 (0.7)	0.29	0.08	0.52
Clarity	6.8 (2.3)	6.8 (2.2)	7.3 (2.2)	0.91	0.09	0.10
Relevance	7.0 (2.2)	7.3 (2.1)	7.6 (2.0)	0.24	0.02	0.24
Quality of distractors	5.8 (2.5)	6.2 (2.2)	6.7 (2.4)	0.24	0.006	0.09
Suitability level *	6.0 (2.3)	6.4 (2.4)	7.0 (2.1)	0.15	<0.001	0.04
Adequate Answers	5.9 (2.4)	6.1 (2.1)	6.6 (2.3)	0.47	0.03	0.10
Difficulty level	5.1 (1.6)	4.9 (1.5)	4.7 (1.5)	0.34	0.06	0.36

Table 1. Legend. The table presents the performance of the two large language models—Google Gemini and ChatGPT—and the physicians’ draft questions across an overall assessment and the six assessment criteria. The table also includes *p*-values comparing the models. Abbreviation: SD, Standard Deviation; * Suitability level is regarding EFSUMB1.

## Data Availability

The original contributions presented in this study are included in the article/Supplementary Material. Further inquiries can be directed to the corresponding author.

## Supplementary Materials

The following supporting information can be downloaded at: https://www.mdpi.com/article/10.3390/diagnostics15151848/s1, File S1: Question Assessment Scales.

## Abbreviations

The following abbreviations are used in this manuscript:

HNUS	Head and neck ultrasound
LLM	Large Language Model
MCQ	Multiple-Choice Questions
EFSUMB	European Federation of Ultrasound Societies in Medicine and Biology
USMLE	United States Medical Licensing Examination
SD	Standard Deviation
ANOVA	Analysis of the Variance
