# Supplementary material for: Evaluation of Multiple-Choice Tests in Head and Neck Ultrasound Created by Physicians and Large Language Models
Source: Diagnostics (Basel). 2025 Jul 22;15(15):1848. doi: 10.3390/diagnostics15151848 (PMC12346108; doi:10.3390/diagnostics15151848)
Supplement: Supplementary file 1 [file diagnostics-15-01848-s001.zip › diagnostics-3746428-supplementary.pdf]

Lymph Nodes

Hi [first_name],Below you will find 30 multiple-choice questions (MCQs) generated by a human, ChatGPT (version GPTo), or Google Gemini (version Gemini Advanced). The chatbots are prompted with the following:"Please create a multiple-choice quiz with 10 questions aimed at evaluating a person's expertise in performing ultrasound examinations on lymph nodes in the ear, nose, and throat regions. The questions should cover various levels of difficulty but must exclude topics related to ultrasound physics. For each question, please provide three answer options including one correct answer, and indicate the level of difficulty (Likert scale from 1-5)." In this way, some questions can be more or less similar. All questions should be evaluated on various aspects using a Likert scale from 1 to 10, except for the question regarding the overall assessment, which should be evaluated using a Likert scale from 1 to 5.For further information or questions please feel free to write an email to: jnie0904@regionh.dkYou can press "Save & Return Later" at the bottom of each page if you want a break. You will then get a link and can continue from where you left off.

Question 1

What is the primary indication for performing an ultrasound examination of lymph nodes in the neck region?

A) Screening for thyroid nodules
B) Evaluation of lymphadenopathy
C) Assessing salivary gland function

Correct Answer: B) Evaluation of lymphadenopathy

1) Clarity of the item, with the stem providing sufficient information to direct a well-prepared candidate towards a single best answer.

Very low clarity — Very high clarity

(Place a mark on the scale above)

2) Relevance of the item to the clinical practice of head and neck ultrasound

Not relevant — Very relevant

(Place a mark on the scale above)

3) Suitability of the item for an EFSUMB level 1 exam based on level of difficulty

Not suitable — Very suitable

(Place a mark on the scale above)

4) Quality of the distractors, which are plausible and similar in concept and structure

Very low quality — Very high quality

(Place a mark on the scale above)

5) Adequacy of the rationale, which appropriately explains why the key is correct and the distractors are incorrect

Not adequate — Highly adequate

(Place a mark on the scale above)

6) How difficult is the question?

Very easy — Very difficult

(Place a mark on the scale above)

7) Overall assessment

Poor — Acceptable — Excellent

(Place a mark on the scale above)

8) Can the question be used directly in a multiple-choice quiz?

- ◯ No
- ◯ Yes, with modifications
- ◯ Yes, without modifications

9) Comments?

____

Question 2

Which of the following lymph node chains is NOT typically assessed in a routine ENT ultrasound examination?

A) Level I (submental and submandibular)
B) Level II (upper jugular)
C) Level V (posterior triangle)

Correct Answer: C) Level V (posterior triangle)

10) Clarity of the item, with the stem providing sufficient information to direct a well-prepared candidate towards a single best answer.

Very low clarity — Very high clarity

(Place a mark on the scale above)

11) Relevance of the item to the clinical practice of head and neck ultrasound

Not relevant — Very relevant

(Place a mark on the scale above)

12) Suitability of the item for an EFSUMB level 1 exam based on level of difficulty

Not suitable — Very suitable

(Place a mark on the scale above)

13) Quality of the distractors, which are plausible and similar in concept and structure

Very low quality — Very high quality

(Place a mark on the scale above)

14) Adequacy of the rationale, which appropriately explains why the key is correct and the distractors are incorrect

Not adequate — Highly adequate

(Place a mark on the scale above)

15) How difficult is the question?

Very easy — Very difficult

(Place a mark on the scale above)

16) Overall assessment

Poor — Acceptable — Excellent

(Place a mark on the scale above)

17) Can the question be used directly in a multiple-choice quiz?

- ◯ No
- ◯ Yes, with modifications
- ◯ Yes, without modifications

18) Comments?

____

Question 3

What is characteristic of normal level 1 lymph nodes in comparison to lymph nodes in levels 2-6?

A) Nodes tend to be rounder
B) Nodes tend to be larger in size
C) Nodes tend to have metastases more frequently

Correct Answer: A) Nodes tend to be rounder

19) Clarity of the item, with the stem providing sufficient information to direct a well-prepared candidate towards a single best answer.

Very low clarity — Very high clarity

(Place a mark on the scale above)

20) Relevance of the item to the clinical practice of head and neck ultrasound

Not relevant — Very relevant

(Place a mark on the scale above)

21) Suitability of the item for an EFSUMB level 1 exam based on level of difficulty

Not suitable — Very suitable

(Place a mark on the scale above)

22) Quality of the distractors, which are plausible and similar in concept and structure

Very low quality — Very high quality

(Place a mark on the scale above)

23) Adequacy of the rationale, which appropriately explains why the key is correct and the distractors are incorrect

Not adequate — Highly adequate

(Place a mark on the scale above)

24) How difficult is the question?

Very easy — Very difficult

(Place a mark on the scale above)

25) Overall assessment

Poor — Acceptable — Excellent

(Place a mark on the scale above)

26) Can the question be used directly in a multiple-choice quiz?

◯ No
◯ Yes, with modifications
◯ Yes, without modifications

27) Comments?

____

Thyroid Gland

Below you will find 30 multiple-choice questions (MCQs) generated by a human, ChatGPT (version GPTo), or Google Gemini (version Gemini Advanced). The chatbots are prompted with the following:"Please create a multiple-choice quiz with 10 questions aimed at evaluating a person's expertise in performing ultrasound examinations on the thyroid gland. The questions should cover various levels of difficulty but must exclude topics related to ultrasound physics. For each question, please provide three answer options including one correct answer, and indicate the level of difficulty (Likert scale from 1-5)." In this way, some questions can be more or less similar. All questions should be evaluated on various aspects using a Likert scale from 1 to 10, except for the question regarding the overall assessment, which should be evaluated using a Likert scale from 1 to 5.For further information or questions please feel free to write an email to: jnie0904@regionh.dk You can press "Save & Return Later" at the bottom of each page if you want a break. You will then get a link and can continue from where you left off.

Question 1

What is the most common indication for performing a thyroid ultrasound?

A) Evaluation of thyroid nodules
B) Screening for thyroid cancer
C) Assessing thyroid hormone levels

Correct Answer: A) Evaluation of thyroid nodules

1) Clarity of the item, with the stem providing sufficient information to direct a well-prepared candidate towards a single best answer.

Very low clarity — Very high clarity

(Place a mark on the scale above)

2) Relevance of the item to the clinical practice of head and neck ultrasound

Not relevant — Very relevant

(Place a mark on the scale above)

3) Suitability of the item for an EFSUMB level 1 exam based on level of difficulty

Not suitable — Very suitable

(Place a mark on the scale above)

4) Quality of the distractors, which are plausible and similar in concept and structure

Very low quality — Very high quality

(Place a mark on the scale above)

5) Adequacy of the rationale, which appropriately explains why the key is correct and the distractors are incorrect

Not adequate — Highly adequate

(Place a mark on the scale above)

6) How difficult is the question?

Very easy — Very difficult

(Place a mark on the scale above)

7) Overall assessment

Poor — Acceptable — Excellent

(Place a mark on the scale above)

8)	Can the question be used directly in a multiple-choice quiz?	◯ No<br>◯ Yes, with modifications<br>◯ Yes, without modifications
9)	Comments?	__________________________

Question 2

Which anatomical structure is most commonly used as a landmark for identifying the thyroid gland on ultrasound?

A) Trachea
B) Jugular vein
C) Carotid artery

Correct Answer: A) Trachea

10) Clarity of the item, with the stem providing sufficient information to direct a well-prepared candidate towards a single best answer.

Very low clarity — Very high clarity

(Place a mark on the scale above)

11) Relevance of the item to the clinical practice of head and neck ultrasound

Not relevant — Very relevant

(Place a mark on the scale above)

12) Suitability of the item for an EFSUMB level 1 exam based on level of difficulty

Not suitable — Very suitable

(Place a mark on the scale above)

13) Quality of the distractors, which are plausible and similar in concept and structure

Very low quality — Very high quality

(Place a mark on the scale above)

14) Adequacy of the rationale, which appropriately explains why the key is correct and the distractors are incorrect

Not adequate — Highly adequate

(Place a mark on the scale above)

15) How difficult is the question?

Very easy — Very difficult

(Place a mark on the scale above)

16) Overall assessment

Poor — Acceptable — Excellent

(Place a mark on the scale above)

17) Can the question be used directly in a multiple-choice quiz?

◯ No
◯ Yes, with modifications
◯ Yes, without modifications

18) Comments?

____

Question 3

Which anatomical landmark is most important for initial localization of the thyroid gland during an ultrasound exam?

A) Cricoid cartilage
B) Hyoid bone
C) Thyroid cartilage

Correct Answer: B) Hyoid bone

19) Clarity of the item, with the stem providing sufficient information to direct a well-prepared candidate towards a single best answer.

Very low clarity — Very high clarity

(Place a mark on the scale above)

20) Relevance of the item to the clinical practice of head and neck ultrasound

Not relevant — Very relevant

(Place a mark on the scale above)

21) Suitability of the item for an EFSUMB level 1 exam based on level of difficulty

Not suitable — Very suitable

(Place a mark on the scale above)

22) Quality of the distractors, which are plausible and similar in concept and structure

Very low quality — Very high quality

(Place a mark on the scale above)

23) Adequacy of the rationale, which appropriately explains why the key is correct and the distractors are incorrect

Not adequate — Highly adequate

(Place a mark on the scale above)

24) How difficult is the question?

Very easy — Very difficult

(Place a mark on the scale above)

25) Overall assessment

Poor — Acceptable — Excellent

(Place a mark on the scale above)

26) Can the question be used directly in a multiple-choice quiz?

◯ No
◯ Yes, with modifications
◯ Yes, without modifications

27) Comments?

____

Salivary Glands

Hi [first_name],Below you will find 30 multiple-choice questions (MCQs) generated by a human, ChatGPT (version GPTo), or Google Gemini (version Gemini Advanced). The chatbots are prompted with the following:"Please create a multiple-choice quiz with 10 questions aimed at evaluating a person's expertise in performing ultrasound examinations on the salivary glands. The questions should cover various levels of difficulty but must exclude topics related to ultrasound physics. For each question, please provide three answer options including one correct answer, and indicate the level of difficulty (Likert scale from 1-5)." In this way, some questions can be more or less similar. All questions should be evaluated on various aspects using a Likert scale from 1 to 10, except for the question regarding the overall assessment, which should be evaluated using a Likert scale from 1 to 5.For further information or questions please feel free to write an email to: jnie0904@regionh.dkYou can press "Save & Return Later" at the bottom of each page if you want a break. You will then get a link and can continue from where you left off.

Question 1

In what salivary gland can you find lymph nodes?

A) The submandibular gland
B) The sublingual gland
C) The parotid gland

Correct Answer: C) The parotid gland

1) Clarity of the item, with the stem providing sufficient information to direct a well-prepared candidate towards a single best answer.

Very low clarity — Very high clarity

(Place a mark on the scale above)

2) Relevance of the item to the clinical practice of head and neck ultrasound

Not relevant — Very relevant

(Place a mark on the scale above)

3) Suitability of the item for an EFSUMB level 1 exam based on level of difficulty

Not suitable — Very suitable

(Place a mark on the scale above)

4) Quality of the distractors, which are plausible and similar in concept and structure

Very low quality — Very high quality

(Place a mark on the scale above)

5) Adequacy of the rationale, which appropriately explains why the key is correct and the distractors are incorrect

Not adequate — Highly adequate

(Place a mark on the scale above)

6) How difficult is the question?

Very easy — Very difficult

(Place a mark on the scale above)

7) Overall assessment

Poor — Acceptable — Excellent

(Place a mark on the scale above)

8) Can the question be used directly in a multiple-choice quiz?

- ◯ No
- ◯ Yes, with modifications
- ◯ Yes, without modifications

9) Comments?

____

Question 2

How can a dilated salivary gland duct be distinguished from a blood vessel?

A) The salivary gland duct will appear without flow on Doppler
B) The salivary gland duct will be bigger than the blood vessel
C) The salivary gland duct will have edge shadowing

Correct Answer: A) The salivary gland duct will appear without flow on Doppler

10) Clarity of the item, with the stem providing sufficient information to direct a well-prepared candidate towards a single best answer.

Very low clarity — Very high clarity

(Place a mark on the scale above)

11) Relevance of the item to the clinical practice of head and neck ultrasound

Not relevant — Very relevant

(Place a mark on the scale above)

12) Suitability of the item for an EFSUMB level 1 exam based on level of difficulty

Not suitable — Very suitable

(Place a mark on the scale above)

13) Quality of the distractors, which are plausible and similar in concept and structure

Very low quality — Very high quality

(Place a mark on the scale above)

14) Adequacy of the rationale, which appropriately explains why the key is correct and the distractors are incorrect

Not adequate — Highly adequate

(Place a mark on the scale above)

15) How difficult is the question?

Very easy — Very difficult

(Place a mark on the scale above)

16) Overall assessment

Poor — Acceptable — Excellent

(Place a mark on the scale above)

17) Can the question be used directly in a multiple-choice quiz?

- ◯ No
- ◯ Yes, with modifications
- ◯ Yes, without modifications

18) Comments?

____

Question 3

Which salivary gland is most commonly affected by sialolithiasis (salivary stones)?

A) Parotid gland
B) Submandibular gland
C) Sublingual gland

Correct Answer: B) Submandibular gland

19) Clarity of the item, with the stem providing sufficient information to direct a well-prepared candidate towards a single best answer.

Very low clarity — Very high clarity

(Place a mark on the scale above)

20) Relevance of the item to the clinical practice of head and neck ultrasound

Not relevant — Very relevant

(Place a mark on the scale above)

21) Suitability of the item for an EFSUMB level 1 exam based on level of difficulty

Not suitable — Very suitable

(Place a mark on the scale above)

22) Quality of the distractors, which are plausible and similar in concept and structure

Very low quality — Very high quality

(Place a mark on the scale above)

23) Adequacy of the rationale, which appropriately explains why the key is correct and the distractors are incorrect

Not adequate — Highly adequate

(Place a mark on the scale above)

24) How difficult is the question?

Very easy — Very difficult

(Place a mark on the scale above)

25) Overall assessment

Poor — Acceptable — Excellent

(Place a mark on the scale above)

26) Can the question be used directly in a multiple-choice quiz?

- ◯ No
- ◯ Yes, with modifications
- ◯ Yes, without modifications

27) Comments?

____
